# Cloning, characterization and expression analysis of porcine microRNAs

**DOI:** 10.1186/1471-2164-10-65

**Published:** 2009-02-05

**Authors:** Alavala Matta Reddy, Yun Zheng, Guru Jagadeeswaran, Simone L Macmil, Wiley B Graham, Bruce A Roe, Udaya Desilva, Weixiong Zhang, Ramanjulu Sunkar

**Affiliations:** 1 Department of Biochemistry and Molecular Biology, Oklahoma State University, Stillwater, OK 74078, USA; 2 Department of Computer Science and Engineering, Washington University in St Louis, St Louis, MO 63130, USA; 3 Department of Chemistry and Biochemistry, University of Oklahoma, 101 David L Boren Blvd, Norman, OK 73019, USA; 4 Department of Animal Sciences, Oklahoma State University, Stillwater, OK 74078, USA; 5 Department of Genetics, Washington University School of Medicine, St Louis, MO 63110, USA

## Abstract

**Background:**

MicroRNAs (miRNAs) are small ~22-nt regulatory RNAs that can silence target genes, by blocking their protein production or degrading the mRNAs. Pig is an important animal in the agriculture industry because of its utility in the meat production. Besides, pig has tremendous biomedical importance as a model organism because of its closer proximity to humans than the mouse model. Several hundreds of miRNAs have been identified from mammals, humans, mice and rats, but little is known about the miRNA component in the pig genome. Here, we adopted an experimental approach to identify conserved and unique miRNAs and characterize their expression patterns in diverse tissues of pig.

**Results:**

By sequencing a small RNA library generated using pooled RNA from the pig heart, liver and thymus; we identified a total of 120 conserved miRNA homologs in pig. Expression analysis of conserved miRNAs in 14 different tissue types revealed heart-specific expression of miR-499 and miR-208 and liver-specific expression of miR-122. Additionally, miR-1 and miR-133 in the heart, miR-181a and miR-142-3p in the thymus, miR-194 in the liver, and miR-143 in the stomach showed the highest levels of expression. miR-22, miR-26b, miR-29c and miR-30c showed ubiquitous expression in diverse tissues. The expression patterns of pig-specific miRNAs also varied among the tissues examined.

**Conclusion:**

Identification of 120 miRNAs and determination of the spatial expression patterns of a sub-set of these in the pig is a valuable resource for molecular biologists, breeders, and biomedical investigators interested in post-transcriptional gene regulation in pig and in related mammals, including humans.

## Background

Spatio-temporal regulation of gene expression is vital for normal growth and development in living organisms and for optimal response to environmental stimuli and endogenous cues. This regulation is achieved by multiple mechanisms operating at transcriptional, post-transcriptional and post-translational levels [1,2]. Transcriptional regulation involving specific transcription factors interacting with their respective DNA *cis*-elements and post-translational modifications involving protein modifications have been known for several decades. Only recently, the discovery of microRNAs (miRNAs; ~22-nt non-coding RNAs) has greatly expanded our knowledge of the cellular mechanisms that regulate gene expression at the post-transcriptional level in eukaryotes [3,4]. Recent studies have suggested that miRNAs could serve as biomarkers for the identification of different types of cancers [5] and/or as therapeutic targets or agents [6,7].

Biochemical studies in animals support a compartmentalized, two-step maturation of miRNAs derived from their precursor transcripts originating from the miRNA genes located in the intergenic regions. Interestingly, many miRNAs in mammals are derived from the introns of the protein coding genes. The miRNAs are loaded into a silencing complex, called RNA-induced silencing complex (RISC) and can guide the complex to the target mRNAs. In most cases, animal miRNAs recognize their target transcripts by base pairing with the 7- to 8-nt complementary region usually located in the 3'UTRs on the target mRNAs and thus repressing their expression [8-10], although mRNA cleavage might also occur [11-13]. In contrast to the well-documented suppression of gene expression by miRNAs, miRNAs were recently suggested to also enhance target gene expression, and this "oscillation of activity between silencers to enhancers of gene expression" appears to depend on the state of the cell cycle [14].

Since the initial discovery that lin-4 acts as a regulatory RNA in *Caenorhabditis elegans *[15], interest in finding miRNAs and understanding their functions in diverse organisms has grown. Thus far, miRNAs have been shown to play critical roles in almost all biological processes examined, such as control of developmental timing, cell proliferation, cell fate specification, embryonic stem cell differentiation, limb development, adipogenesis, myogenesis, angiogenesis and hematopoiesis, neurogenesis, apoptosis, fat metabolism, insulin secretion, and even cancer [4,16]. Many miRNA families are conserved among the vertebrate animals, and their functions may also have been well conserved. However, many of the new miRNAs recently discovered in human and chimpanzee are not conserved beyond mammals, and ~10% are taxon specific [17]. As a result of extending this type of studies to more animal species, a number of lineage-specific miRNAs [18,19] and species-specific miRNAs [18,20,21] have been identified. These findings suggested that sequencing small RNA libraries from individual organisms is important to identify and catalog conserved and novel species-specific miRNAs. Computational approaches are effective in identifying conserved miRNAs in diverse plant or animal species [22,23]. However, the process requires knowledge of the complete genome sequence, and species-specific miRNAs cannot be identified with confidence without this information. Direct small RNA sequencing is a straightforward and effective approach to characterize the miRNAs expressed from a genome of an organism [24].

Pork, derived from pigs (*Sus scrofa*), is one of the most widely eaten meats in the world [25,26]. In recent years, the pig has been recognized as a potential model system for biomedical research, because pigs and humans have similarities in many aspects of their anatomy, physiology, biochemistry, pathology and pharmacology [25,26]. Consequently, pigs can offer a system for understanding an array of health-related aspects of humans, such as obesity, diabetes, cancer, gastric ulcers, female reproductive health, cardiovascular disease, infectious diseases and organ transplantation [25]. Additionally, pigs are closer evolutionarily to humans than mice [25,27]. The economic and biomedical significance of pigs has led to the launch of the Swine Genome Sequencing Consortium (SGSC) [28] to decipher the genome of the pig. The availability of the genome sequence and transcriptome analyses would significantly advance our ability to decipher various biological and biomedical secrets for better exploitation of the commercial traits of pigs as well as for the benefit of human health [26]. Identification of miRNAs and their target genes can provide further insights into the post-transcriptional gene regulatory mechanisms influencing various biological and metabolic processes in pigs.

Bioinformatic approaches have been used previously to identify miRNAs in pig [25] by exploiting the available genome sequence, 55 conserved miRNAs have been predicted (latest miRBase release 11.1 April, 2008). Recently, Kim et al. [29] reported the identification of 17 new miRNAs belonging to conserved miRNA families in pig. However, this number is small compared to the several hundreds of conserved miRNAs known across the animal kingdom. In this study, we sequenced a small RNA library and experimentally validated 120 miRNAs. Only 24 matched the pig miRNAs listed in the miRBase (Table 1). The remaining 96 miRNAs represent new miRNA homologs belonging to conserved miRNA families (Table 2). Furthermore, we determined the temporal expression of 22 conserved and four pig-specific miRNAs in diverse tissues of pig.

**Table 1:** Expression-based confirmation of previously predicted miRNAs in pig.

**miRNA**	**Sequence**	**Frequency**	**Conservation**
ssc-let-7c	UGAGGUAGUAGGUUGUAUGGUU	63	hsa, mmu, rno, gga, dre, xtr, sme, bta, odi, cin, csa, mml, cfa

ssc-let-7f	UGAGGUAGUAGAUUGUAUAGUU	18	hsa, mmu, rno, gga, dre, xtr, bta, mdo, mml, cfa

ssc-let-7i	UGAGGUAGUAGUUUGUGCU	9	mmu, hsa, rno, gga, dre, fru, tni, xtr, bta, mdo, mml

ssc-miR-106a	AAAAGUGCUUACAGUGCAGGUAGC	2	hsa, mmu, ggo, age, ppa, mml, ppy, ptr, sla, mne, cfa

ssc-miR-122	UGGAGUGUGACAAUGGUGUUUGU	126	bta, mmu, hsa, rno, gga, dre, fru, tni, xtr, mdo, mml

ssc-miR-124a	UUAAGGCACGCGGUGAAUGCCA	1	gga, ggo, age, ppa, ppy, ptr, mml, lla, bta, sme, mdo, odi, mmu

ssc-miR-125b	UCCCUGAGACCCUAACUUGUGA	2	mmu, hsa, rno, gga, dre, ggo, age, ppa, ppy, ptr, mml, sla, lla, mne, lca, tni, bta, xtr, sme, mdo, bta, cfa

ssc-miR-128	UCACAGUGAACCGGUCUCUUUU	4	mmu, hsa, rno, gga, dre, mml, ptr, ppy, sla, age, ppa, fru, tni, bta, xtr, mdo, mml, cfa

ssc-miR-145	GUCCAGUUUUCCCAGGAAUCCCUU	1	hsa, mmu, rno, dre, mml, ptr, ggo, ppy, mne, bta, xtr, mdo, dre

ssc-miR-148a	UCAGUGCACUACAGAACUUUGU	1	hsa, mmu, gga, bta, xtr, mml, cfa

ssc-miR-15b	CCGCAGCACAUCAUGGUUUACA	4	hsa, mmu, rno, gga, dre, ggo, age, ppa, ppy, ptr, mml, lla, mne, fru, tni, xtr, bta, cfa

ssc-miR-181b	AACAUUCAUUGCUGUCGGUGGGUU	1	hsa, mmu, rno, gga, dre, mml, ptr, ppy, ggo, lla, mne, ppa, fru, tni, xtr, bta, mdo, mml, cfa

ssc-miR-181c	AACAUUCAACCUGUCGGUGAGU	1	hsa, mmu, rno, dre, mml, ptr, ggo, ppa, bta, cfa, mdo

ssc-miR-20	UAAAGUGCUUAUAGUGCAGGUA	9	mmu, rno, gga, gga, hsa, dre, bta, xtr, mml, xla, ggo, lca, age, ppa, ppy, ptr, sla, lla, mne, fru, tni, mdo, cfa

ssc-miR-21	UAGCUUAUCAGACUGAUGUUGA	4	hsa, mmu, rno, dre, mml, ptr, ggo, ppy, mne, age, ppa, fru, tni, bta, gga, mdo, cgr, cfa

ssc-miR-23a	AUCACAUUGCCAGGGAUUUCC	1	hsa, mmu, rno, dre, ggo, age, ppa, lca, ppy, ptr, mml, sla, mne, fru, tni, xtr, bta, mdo, cfa

ssc-miR-24	UGGCUCAGUUCAGCAGGAACAG	3	xtr, hsa, mmu, rno, gga, dre, ppy, mne, ppa, ggo, ptr, mml, fru, tni, bta, mdo, cfa

ssc-miR-26a	UUCAAGUAAUCCAGGAUAGGCU	93	mmu, has, mmu, rno, gga, dre, ptr, ggo, ppy, lla, mne, mml, ppa, bta, mml, cfa, dre

ssc-miR-27a	UUCACAGUGGCUAAGUUCCGC	4	hsa, mmu, rno, dre, ggo, age, ppa, lca, ppy, ptr, mml, sla, mne, bta, xtr, mdo, cfa

ssc-miR-29b	UAGCACCAUUUGAAAUCAGU	6	hsa, mmu, rno, gga, dre, ppy, ptr, ggo, lla, age, ppa, ptr, ggo, ppy, sla, mne, ppa, fru, tni, xtr, bta, mdo, ame, mml, cfa

ssc-miR-29c	UAGCACCAUUUGAAAUCGGUUA	6	mmu, hsa, rno, gga, xtr, bta, mml, cfa

ssc-miR-30c	UGUAAACAUCCUACACUCUCAGC	3	hsa, mmu, bta, mmu, rno, gga, dre, ptr, lla, mne, fru, tni, xtr, mml, cfa

ame: *Apis mellifera*, aga: *Anopheles gambiae*, age: *Ateles geoffroyi*, bmo: *Bombyx mori*, bta: *Bos taurus*, cbr: *Caenorhabditis briggsae*, cel: *Caenorhabditis elegans*, cfa: *Canis familiaris*, cgr: *Cricetulus griseus*, cin: *Ciona intestinalis*, csa: *Ciona savignyi*, dme: *Drosophila melanogaster*, dps: *Drosophila pseudoobscura*, dre: *Danio rerio*, fru: *Fugu rubripes*, gga: *Gallus gallus*, ggo: *Gorilla gorilla*, hsa: *Homo sapiens*, lca: *Lemur catta*, lla: *Lagothrix lagotricha*, mdo: *Monodelphis domestica*, mml: *Macaca mulatta*, mmu: *Mus musculus*, mne: *Macaca nemestrina*, odi: *Oikopleura dioica*, ppa: *Pan paniscus*, ppy: *Pongo pygmaeus*, ptr: *Pan troglodytes*, rno: *Rattus norvegicus*, sla: *Saguinus labiatus*, sme: *Schmidtea mediterranea*, ssc: *Sus scrofa*; tni: *Tetraodon nigroviridis*, xla: *Xenopus laevis*, xtr: *Xenopus tropicalis*

**Table 2:** Newly identified miRNAs in pig that are homologous to known miRNAs from other animal species.

**miRNA**	**Sequence**	**Frequency**	**Conservation**
ssc-miR-1a	UGGAAUGUAAAGAAGUAUGUA	317	gga, xtr, sme, odi

ssc-miR-1b	UGGAAUGUCGUGAAUUAUGGUC	22	gga, xtr, sme, odi, mmu, hsa

ssc-miR-1c	UGGAAUGUAAAGAAGUAUGUGA	72	sme, odi

ssc-let-7a	UGAGGUAGUAGGUUGUAUAGUU	80	hsa, mmu, rno, gga, dre, fru, tni, xtr, bta, sme, mdo, odi, cin, csa, mml, cfa

ssc-let-7b	UGAGGUAGUAGUUUGUGUAGUU	64	hsa, mmu, rno, gga, dre, fru, tni, xtr, bta, sme, mdo, odi, cin, csa, mml

ssc-let-7d	UGAGGUAGUUGGUUGUAUUGUU	32	hsa, mmu, rno, gga, dre, fru, tni, bta, mdo, odi, cin, csa, mml

ssc-let-7e	UGAGGUAGUAGGUUGUUUAGUU	63	hsa, mmu, rno, dre, fru, tni, xtr, bta, cin, mml, cfa

ssc-let-7g	UGAGGUAGUAGUUUGUACAGU	25	hsa, mmu, gga, dre, fru, tni, xtr, bta, mdo, mml, cfa

ssc-let-7h	UGAGGUAGUAAGUUGUGUUGUU	5	dre, fru, tni

ssc-let-7j	UGAGGUAGUAGAGUGCAGUAGUU	80	gga, fru, tni, dre, cfa

ssc-let-7k	UGAGGUAGUAGAUUGAAUAGUU	6	gga

ssc-miR-10a	UACCCCGUAGAUCCGAAUUUGUG	1	hsa, mmu, rno, dre, ggo, ppy, sla, age, ppa, xtr, bta, mdo, mml, mmu

ssc-miR-15a	UAGCAGCACGGAAUGGUUUGUG	2	hsa, mmu, gga, dre, age, ggo, mne, sla, ppa, lca, mml, ppy, ptr, lla, fru, tni, xtr, mdo, bta, cfa

ssc-miR-15c	AAGCAGCGCGUCAUGGUUUUC	4	dre, xtr

ssc-miR-16a	UAGCAGCACGUAAAUAUUGGUG	5	dre, xtr

ssc-miR-16b	UAGCAGCACGUAAAUAUUGGAG	5	dre, xtr

ssc-miR-16c	UAGCAGCACGUAAAUACUGGAG	4	dre, xtr

ssc-miR-17	CAAAGUGCUUACAGUGCAGGUA	2	dre, hsa, mmu, rno, gga, ggo, lca, age, ppa, ppy, ptr, mml, sla, lla, mne, fru, tni, xtr, bta, mdo, rno, cfa

ssc-miR-18a	UAAGGUGCAUCUAGUGCAGAUA	4	hsa, mmu, rno, gga, dre, bta, xtr

ssc-miR-18b	UAAGGUGCAUCUAGUGCAGUUA	5	gga, hsa, dre, bta, xtr, mmu, mml

ssc-miR-20a	UAAAGUGCUUAUAGUGCAGGUAG	10	hsa, mmu, rno, gga, dre, mml, bta, xtr

ssc-miR-20b	CAAAGUGCUCACAGUGCAGGUA	3	hsa, mmu, rno, gga, dre, mml, bta, xtr

ssc-miR-22a	AAGCUGCCAGCUGAAGAACUGU	1	dre, fru, tni

ssc-miR-22b	AAGCUGCCAGUUGAAGAGCUGU	5	dre, fru, tni

ssc-miR-23b	AUCACAUUGCCAGGGAUUACCAC	1	mmu, hsa, rno, gga, dre, ptr, ppy, ppa, fru, tni, xtr, bta, mdo, mml, cfa

ssc-miR-24a	UGGCUCAGUUCAGCAGGAACAG	3	xtr

ssc-miR-24b	UGGCUCAGUUCAGCAGGACAG	2	xtr

ssc-miR-25	CAUUGCACUUGUCUCGGCUGA	1	cel, cbr, dme, hsa, mmu, rno, dre, ggo, ppa, ppy, ptr, mml, lla, mne, fru, tni, xtr, bta, mdo, cfa

ssc-miR-26b	UUCAAGUAAUCCAGGAUAGGUU	84	hsa, mmu, rno, dre, bta, mml, cfa

ssc-miR-27b	UUCACAGUGGCUAAGUUCUGC	4	hsa, mmu, rno, gga, dre, fru, tni, bta, xtr, mdo, mml, cfa

ssc-miR-27c	UUCACAGUGGCUAAGUUCCAC	4	dre, fru, tni, xtr

ssc-miR-27d	UUCACAGUGGCUAAGUUCUUCA	4	dre

ssc-miR-27e	UUCACAGUGGCUAAGUUCAGUG	4	dre, fru, tni

ssc-miR-29a	CUAGCACCAUCUGAAAUCGGUU	6	hsa, mmu, rno, gga, dre, ggo, age, ppa, ppy, ptr, mml, sla, lla, mne, fru, tni, bta, xtr, mdo, cfa

ssc-miR-30a	UGUAAACAUCCUCGACUGGAAG	2	hsa, mmu, rno, gga, dre, mml, ptr, ggo, ppy, ppa, xtr, bta, mdo, cfa

ssc-miR-30b	UGUAAACAUCCUACACUCAGC	3	mmu, hsa, rno, gga, dre, mml, ptr, ggo, lla, mne, age, ppa, fru, tni, bta, xtr, cfa

ssc-miR-30d	UGUAAACAUCCCCGACUGGAAG	1	hsa, mmu, rno, gga, dre, ptr, ggo, mne, ppa, fru, tni, bta, xtr, mml, cfa

ssc-miR-30e	CUUUCAGUCGGAUGUUUACAGC	1	hsa, mmu, rno, gga, dre, xtr, bta, mml, cfa

ssc-miR-92a	UAUUGCACUCGUCCCGGCCUUG	2	hsa, dme, mmu, rno, dps, aga, dre, mml, xtr, ame, odi, cin, csa, mml, cfa

ssc-miR-92b	AAUUGCACUAGUCCCGGCCUGC	2	dme, dps, aga, hsa, xtr, mmu, rno, odi, cin, csa, mml, cfa

ssc-miR-93a	AAAGUGCUGUUCGUGCAGGUAG	5	xtr

ssc-miR-93b	AAGUGCUGUUCGUGCAGGUAG	5	xtr

ssc-miR-98	UGAGGUAGUAAGUUGUAUUGUU	5	hsa, mmu, rno, mml, ptr, ggo, ppy, age, ppa, xtr, bta, cfa

ssc-miR-99	AACCCGUAGAUCCGAUCUUGUG	2	hsa, mmu, rno, gga, mml, ptr, ggo, ppy, lla, mne, ppa, bta, mml, cfa, dre, xtr

ssc-miR-100	AACCCGUAAUUCCGAACUUGUG	3	hsa, dme, mmu, rno, gga, dps, aga, dre, ggo, age, ppa, ppy, ptr, mml, sla, lla, fru, tni, xtr, mdo, ame

ssc-miR-101a	UACAGUACUGUGAUAACUGAA	8	mmu, rno, dre, fru, tni, xtr

ssc-miR-101b	UACAGUACUAUGAUAACUGAAG	5	rno, mmu, dre, fru, tni

ssc-miR-106	AAAAGUGCUUAUAGUGCAGGUAGA	10	gga, xtr, bta

ssc-miR-106b	UAAAGUGCUGACAGUGCAGAU	1	mmu, hsa, rno, ggo, age, ppa, ppy, ptr, mml, sla, lla, mne, cfa

ssc-miR-124	UAAGGCACGCGGUGAAUGCCA	1	mmu, dme, hsa, cel, cbr, rno, gga, dps, ame, aga, dre, ggo, age, ppa, ppy, ptr, mml, lla, fru, tni, xtr, bmo, bta, sme, odi, cin, csa, cfa

ssc-miR-125c	UCCCUGAGACCCUAACUCGUGA	2	dre

ssc-miR-126	CAUUAUUACUUUUGGUACGCG	22	mmu, hsa, rno, gga, dre, fru, tni, bta, xtr, cin, csa, mml, cfa

ssc-miR-1297	UUCAAGUAAUUCAGGUG	20	hsa

ssc-miR-130a	CAGUGCAAUGUAAAAAGGGCAU	5	hsa, mmu, rno, gga, dre, mml, ggo, mne, ppa, xtr, mdo, cfa

ssc-miR-130c	CAGUGCAAUAUUAAAAGGGCAU	1	dre, xtr

ssc-miR-133a	UUGGUCCCCUUCAACCAGCUG	2	mmu, hsa, rno, gga, xla, dre, ggo, age, ppa, ppy, ptr, mml, sla, lla, mne, xtr, mdo, mmu

ssc-miR-133b	UUGGUCCCCUUCAACCAGCUA	2	mmu, hsa, gga, dre, rno, xtr, mml

ssc-miR-133c	UUUGGUCCCUUUCAACCAGCUA	2	gga, dre, xtr, mml

ssc-miR-133d	UUGGUCCCCUUCAACCAGCCGC	1	xtr

ssc-miR-141	UAACACUGUCUGGUAACGAUGC	1	hsa, mmu, rno, dre, mml, ggo, ppy, ppa, mdo, cin, csa

ssc-miR-142	CAUAAAGUAGAAAGCACUAC	7	hsa, bta, mmu, rno, gga, dre, fru, tni, xtr, mdo, mml, cfa

ssc-miR-142-3p	UGUAGUGUUUCCUACUUUAUGG	6	mmu, hsa, rno, gga, xtr, mml

ssc-miR-142a	CAUAAAGUAGAAAGCACUACU	3	dre, fru, tni

ssc-miR-143	UGAGAUGAAGCACUGUAGCUCG	14	hsa, dre, mmu, rno, ptr, ggo, ppy, lla, ppa, xtr, mdo, mml, cfa, cbr

ssc-miR-144	UACAGUAUAGAUGAUGUACU	6	mmu, hsa, rno, dre, ptr, ppy, mne, ppa, fru, tni, xtr, gga, mdo, mml, cfa

ssc-miR-146a	UGAGAACUGAAUUCCAUAGAUGG	2	mmu, hsa, rno, dre, mml, cfa

ssc-miR-146b	UGAGAACUGAAUUCCAUAGGCG	2	dre, hsa, gga, mmu, xtr, rno, mml, cfa

ssc-miR-181a	AACAUUCAACGCUGUCGGUGAG	5	mmu, hsa, rno, gga, dre, ggo, ppa, ptr, mml, sla, mne, ppy, lla, fru, bta, xtr, mdo, cfa, tni

ssc-miR-192	CUGACCUAUGAAUUGACAGCCAG	2	hsa, mmu, rno, dre, fru, tni, xtr, bta, mml, cfa

ssc-miR-194	UGUAACAGCAACUCCAUGUGG	3	mmu, hsa, rno, gga, dre, mml, ptr, ppy, ggo, mne, age, fru, tni, xtr, cfa

ssc-miR-197	UUCACCACCUUCUCCACCCAGC	1	hsa, ptr, ppy, mne, age, ppa, mmu, mml, cfa

ssc-miR-200a	UAACACUGUCUGGUAACGAUGU	1	hsa, mmu, rno, gga, dre, fru, tni, xtr, bta, mdo, mml

ssc-miR-204a	UUCCCUUUGUCAUCCUAUGCCU	2	tni

miR-204b	UUCCCUUUGUUAUCCUAUGCCU	1	tni, fru

ssc-miR-211	UUCCCUUUGUCAUCCUUCGCCU	2	mmu, hsa, rno, mml, ppy, mne, gga

ssc-miR-215	AUGACCUAUGAAUUGACAGAC	2	mmu, hsa, gga, mml, ptr, ppy, ggo, mne, rno

ssc-miR-320a	AAAAGCUGGGUUGAGAGGGCGA	1	hsa

ssc-miR-320b	AAAAGCUGGGUUGAGAGGGCAA	1	hsa

ssc-miR-320c	AAAAGCUGGGUUGAGAGGGU	1	hsa

ssc-miR-320d	AAAAGCUGGGUUGAGAGGA	1	hsa

ssc-miR-352	AGAGUAGUAGGUUGCAUAGUA	10	rno

ssc-miR-374a	UUAUAAUACAACCUGAUAAGU	1	hsa, mml, cfa

ssc-miR-378	ACUGGACUUGGAGUCAGAAGGC	11	hsa, mmu, rno, mml, cfa

ssc-miR-450a	UUUUGCGAUGUGUUCCUAAUAU	1	hsa, mmu, rno, mmu, mml, cfa

ssc-miR-451	AAACCGUUACCAUUACUGAGUUU	1	hsa, mmu, rno, dre, xtr, gga, mdo, mml

ssc-miR-455-3p	GCAGUCCAUGGGCAUAUACAC	1	hsa, mml

ssc-miR-499	UUAAGACUUGCAGUGAUGUUU	1	hsa, gga, rno, mmu, bta, dre, xtr, mml, cfa

ssc-miR-503	UAGCAGCGGGAACAGUACUGCAG	1	hsa, mmu, rno, mml, cfa

ssc-miR-739	AGGCCGAAGUGGAGAAGGGUU	3	dre

ssc-miR-891b	UGCAACUUACCUGAGUCAUUGA	1	hsa

ssc-miR-1224-3p	CCCCACCUCCUCUCUCCUCAG	1	hsa, ptr

ame: *Apis mellifera*, aga: *Anopheles gambiae*, age: *Ateles geoffroyi*, bmo: *Bombyx mori*, bta: *Bos taurus*, cbr: *Caenorhabditis briggsae*, cel: *Caenorhabditis elegans*, cfa: *Canis familiaris*, cin: *Ciona intestinalis*, csa: *Ciona savignyi*, dme: *Drosophila melanogaster*, dps: *Drosophila pseudoobscura*, dre: *Danio rerio*, fru: *Fugu rubripes*, gga: *Gallus gallus*, ggo: *Gorilla gorilla*, hsa: *Homo sapiens*, lca: *Lemur catta*, lla: *Lagothrix lagotricha*, mdo: *Monodelphis domestica*, mml: *Macaca mulatta*, mmu: *Mus musculus*, mne: *Macaca nemestrina*, odi: *Oikopleura dioica*, ppa: *Pan paniscus*, ppy: *Pongo pygmaeus*, ptr: *Pan troglodytes*, rno: *Rattus norvegicus*, sla: *Saguinus labiatus*, sme: *Schmidtea mediterranea*, ssc: *Sus scrofa*; tni: *Tetraodon nigroviridis*, xtr: *Xenopus tropicalis*

## Results

### miRNA library construction with pooled RNA from the heart, thymus and liver of pig

Recent systematic analysis of the spatial expression of miRNAs in zebra fish larvae showed that most tissues and organs express a unique miRNA complement [30]. To identify conserved and pig-specific miRNAs, we constructed a small RNA library using pooled RNA from the heart, thymus and liver of the pig. Heart represents a muscle tissue with profound diversity in gene expression [26], thymus is a vital part of the immune system essential for T-lymphocytes development and maturation, and liver represents a tissue with specialized and restricted function and was found to have low level of transcript diversity [26]. The miRNAs from such a library should reflect a higher abundance of heart-, liver- and thymus-specific miRNAs. Additionally, the library should provide an opportunity to identify miRNAs expressed with low-abundance in these tissues. Small RNAs (15–30 nt) were recovered by size fractionation, and the library was constructed as described previously [31-33].

### Sequence analysis and identification of conserved miRNA homologs in the pig

**Figure 1:**
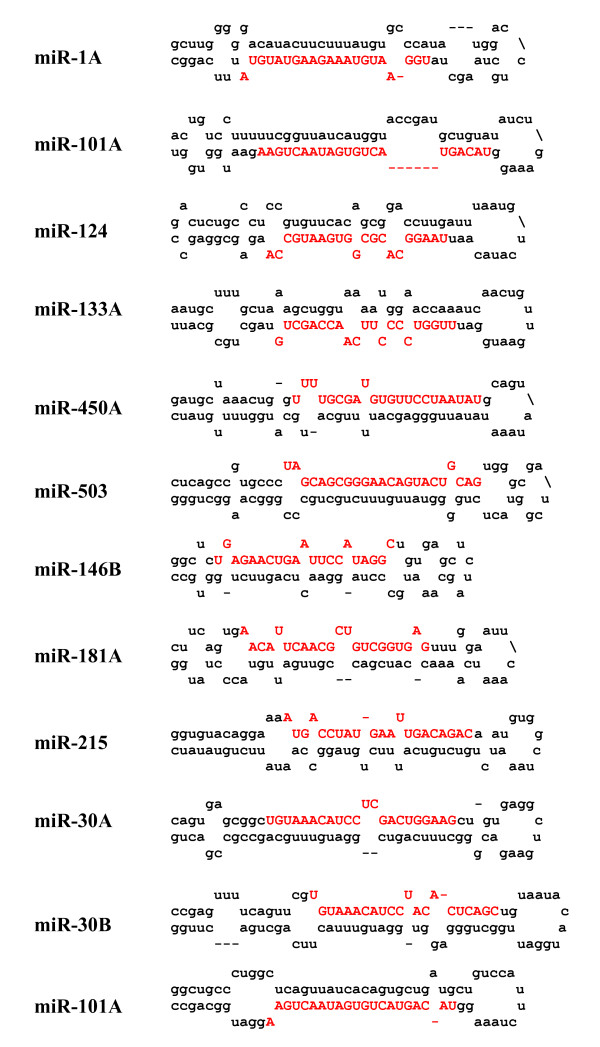
**Predicted fold-back structures using the conserved miRNA homolog precursor sequences from the procine**.

Of the 20,000 reads obtained from the 454/Roche GS-FLX massively parallel pyrosequencer, ~12,592 quality small RNA sequences ranging from 15 to 30-nt were extracted after removal of the 5' and 3' adapters [see Additional file 1]. The quality of our small RNA library is poor as reflected by the accumulation of degradation products from miRNAs (shorter sequences ranging in size between 15 to 8-nt). While the sequencing depth obtained in this study is certainly not exhaustive, but these many reads should be sufficient to identify many of the novel but conserved miRNA homologs in pig. The low depth of the library could also be due to the technical problems associated with the sequencing. All identical sequences were counted and eliminated from the dataset, and only unique sequences with associated read counts were analyzed further. These unique sequences were subjected to BLAST analysis against the Rfam database to remove the ribosomal RNA, tRNA and snRNA breakdown products. This analysis removed ~304 sequences that represented largely breakdown products of rRNAs and tRNAs (Table 3). A search against RepBase revealed 91 sequences as possible sequences derived from repeats. Thus far, miRBase lists only 55 pig miRNAs that were computationally predicted. A BLAST search with the use of the remaining unique small RNA sequences revealed 24 unique sequences (368 reads) that exactly matched with the pig miRNAs listed in the latest miRBase release 11.0 (April, 2008) (Table 1). Our identification of less than 50% of the pig miRNAs listed in miRBase could be due to the use of highly specialized tissues (heart, thymus and liver) for miRNA library construction. BLAST searches against miRBase were performed with remaining small RNAs to identify pig counterparts of the conserved miRNAs. This analysis revealed 98 new miRNAs (represented by 1158 reads) that are homologs of the conserved miRNAs (Table 2 and Figure 1). Together, our study confirmed the expression of 120 conserved miRNAs (24 predicted miRNAs deposited in the miRBase and 96 new miRNAs) in pig (Tables 1 and 2). A significant number (6728) of small RNAs could not be mapped to the available pig genome as it exists at present. A small portion of these sequences could be potentially pig-specific miRNAs or other important small RNAs but their annotation requires the complete genome sequence.

**Table 3:** Distribution of small RNA reads in the sequenced library.

**Small RNA category**	**Number of reads**
rRNA, tRNA, snRNA, etc.	304

miRBase	1562

mRNAs	3907

RepBase	91

Number of reads mapped to the genomic sequences	5864

Number of reads could not be mapped to the genomic sequences	6728

Total number of reads obtained	12592

In the present study, the cloning frequency of newly identified miRNAs varied greatly (1 to > 400) (Tables 1 and 2). Some of the sequences had slight-shifts in their 5' and 3' ends, which could be attributed to processing shifts or enzymatic modifications of miRNAs such as RNA editing [34], 3' nucleotide additions [35] or sequencing artifacts.

### Abundantly expressed miRNA families in the heart, liver and thymus: Sequence-based analysis

**Figure 2:**
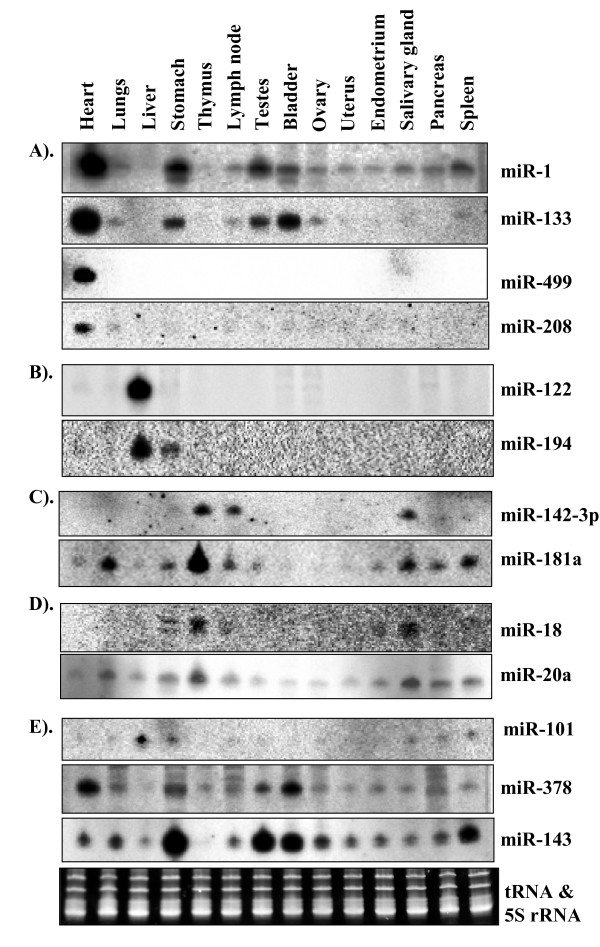
**Expression analysis of conserved miRNAs in different porcine tissues**. A). Heart-specific miRNAs or miRNAs abundantly expressed in the heart, B) Liver-specific miRNAs or miRNAs abundantly expressed in the liver, C) miRNAs showing strong expression in the thymus, D) Expression analysis of miR-18a and miR-20a, the miRNAs located in the miR-17-92 cluster, and E). miR-101, miR-378 and 143 expression patterns.

**Figure 3:**
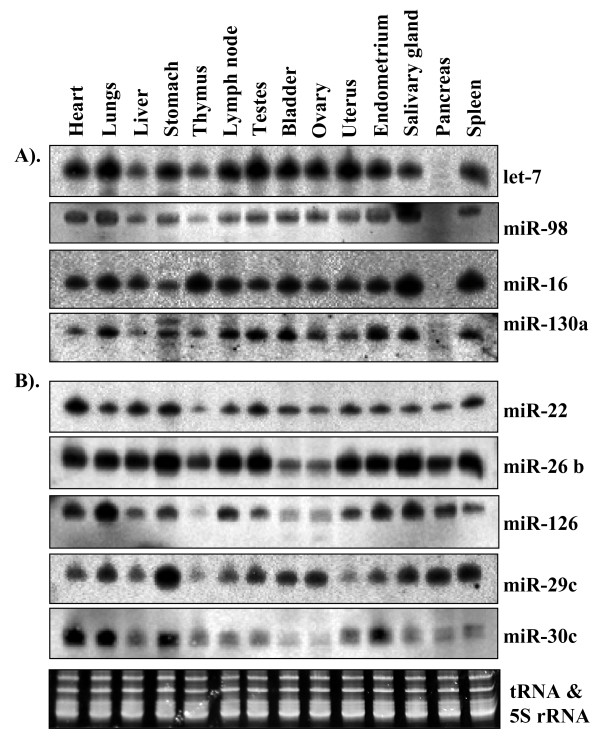
**Expression analysis of ubiquitously and uniformly expressed miRNAs in different porcine tissues**. A) miRNAs expressed in all tissues, but not in the pancreas, B) miRNAs expressed in all 14 tissues tested.

Because, we pooled the total RNA from the heart, liver and thymus for library construction, matching miRNAs to tissue sources is not possible. Nevertheless, by comparing the tissue-specific expression patterns represented by the sequence counts, we can suggest the relative tissue contribution of at least a few miRNA families. For instance, the miR-1 family has the highest frequency (411 times) in our sequences (Table 2) and the highest level of expression in the heart, but was barely detected in thymus and liver (Figure 2). Therefore the total miR-1 count in our sequences could be derived largely from heart tissue. As another example, miR-122 is represented by 126 reads (Table 1). It is a liver-specific miRNA, as determined by a small RNA blot analysis (Figure 2B; [36], and therefore, its total count could be attributed to the liver source. Some other miRNAs are ubiquitously expressed in the heart, liver and thymus, so their counts in our sequences could be attributed to the 3 tissue sources. For instance, let-7 is represented by 445 reads and miR-26 by 177 reads (Tables 1 and 2), and these two miRNAs are ubiquitously expressed in the heart, liver and thymus (Figure 3A and 3B). Thus, miRNA families (e.g., miR-1 and miR-122) that are specifically or highly expressed in any one of the 3 tissues, or miRNAs that are expressed ubiquitously (e.g., let-7 and miR-26) in all 3 tissues, show a far greater frequency than other miRNAs. A few notable exceptions are miR-499, an miRNA abundantly expressed in the heart (Figure 2A), which is represented by only one read (Table 2), and the miR-133 family, which is preferentially and abundantly expressed in the heart (Figure 2), and represented by only 7 reads (Table 1). These observations suggest that cloning frequency does not always reflect the true abundance. This could be attributed to the biased cloning, although what causes the biased cloning of small RNAs is unknown.

In animals and plants, miRNAs exist as multigene families (two or more closely related sequences and vary by 1 or 2 nt only). With use of small RNA blot analysis or probe-based miRNA micro array, discriminating individual members within a miRNA family is difficult. The expression information of a member of a miRNA family is useful for understanding its functions and for practical use for gene manipulations [37]. The cloning frequency-based determination of miRNA abundance can be used to assess the relative expression of each member within a miRNA family. The miR-1 family is represented by three members (miR-1a, miR-1b and miR-1c) in diverse animals (miRBase). We cannot ascertain whether the miR-1 family is also represented by three members in pig because of the lack of complete genome information, but is possible because we found miR-1a, miR-1b and miR-1c homologs in our library (Table 2). Of these three members, miR-1a and miR-1c are represented by 312 and 72 reads, respectively, whereas miR-1b is represented by a lower number (22 reads). The let-7 family has 10 members in diverse animal species (miRBase). Here, we found evidence for the expression of all 10 let-7 members in pig. Interestingly, the expression abundance varies among the let-7 family members (Tables 1 and 2); let-7a and let-7j, each have 80 reads; similarly, let-7b, let-7c and let-7e have almost the same number of reads (63–64); let-7d, let-7f and let-7j have 18 to 32 reads; and let-7h, let-7i and let-7k have a lower number of reads (5–9) (Tables 1 and 2). The miR-98 sequence differs from that of the let-7 family by one nt at position 11 from the 5' end, thus miR-98 is also a member of the let-7 family. miR-98 is represented by 5 reads in our sequences (Table 2). Hence the let-7 miRNA family is represented by 11 members, and this study provides the evidence for the expression of all 11 let-7 family members in pig. Similarly, we found all members of the miR-15, miR-16, miR-18 and miR-133 families in our sequences, suggesting that all members belonging to these miRNA families are expressed in these three (heart, liver and thymus) tissues.

Several recent studies found a number of lineage-specific miRNAs [18,19] or species-specific miRNAs [18,20,21]. Small RNAs in our library lacking annotations in miRBase but having perfect matches to the available pig genome are novel miRNA candidates. Some of the conserved miRNA homologs had appeared only once in our sequences (Table 1 and 2). Several candidate miRNAs are identified and some of them could be even detected using small RNA blot analysis (data not shown). These sequences have several loci in the pig genome. Fold-back structures could be predicted for some loci, but not for all. However, these evidences are not adequate to annotate them as miRNAs.

### Expression analysis of miRNAs in pig

#### Tissue-specific expression of miRNAs in pig

The expression levels of individual miRNAs can be determined by small RNA blot analysis [38,39]. The expression of miRNAs is tightly regulated, both in time and space. To analyze the tissue-specific expression patterns of miRNAs, we analyzed 14 diverse tissues: ovary, heart, salivary glands, liver, lung, pancreas, stomach, lymph node, spleen, uterus, endometrium, pancreas, bladder and thymus. Tissues were collected and stored at -80°C by the DeSilva laboratory as part of the ongoing porcine gene expression project [40]. A recent study revealed the expression of a high proportion of functionally unaccountable genes in pig muscle [26], which forms the basis for analyzing miRNA expression analyses in the muscle tissue such as the heart, stomach and bladder. Liver and lung are tissues with specialized and restricted function and have a low level of transcript diversity [26], so we examined the miRNA expression in these tissues. We chose testes as representative of male reproductory tissues. Interestingly, greater gene diversity in testes has been reported [26]. Uterus, ovaries and endometrium were chosen for miRNA expression analysis as representatives of female reproductive tissues. Immune-related tissues were lymph node and thymus. We also included specialized organs such as pancreas, spleen and salivary glands in the analysis.

#### miRNAs abundantly or specifically expressed in the heart

miR-1 is one of the highly conserved miRNAs and found to be abundantly and specifically expressed in the heart and other muscular tissues [41,42]. Our small RNA blot analysis indicated that miR-1 was highly expressed in the heart but moderately in the stomach, testes, bladder and spleen (Figure 2). miR-1 was barely detected in the liver, with only trace amounts in the thymus (Figure 2). Thus, the high abundance of miR-1 as indicated by the number of sequence reads is associated with its high expression in the heart. The high level of miR-1 in the pig heart is in agreement with previous reports [43,44]. Like miR-1, miR-133 is a muscle-specific miRNA (Figure 2) because of its abundant expression in many other muscular tissues such as heart and skeletal muscle [45,46]. The expression patterns of miR-1 and miR-133 largely overlapped in many tissues examined in this study (Figure 2).

Because of their location within the introns of myosin genes and their specific expression in myogenic cells, miR-208 and miR-499 were referred to as MyomiRs [47]. miR-208 is encoded in intron 27 of the human and mouse αMHC gene [48]. Because this is an intronic-derived miRNA, its expression pattern is similar to that of the host gene, αMyosin Heavy Chain (αMHC) or Myh6. αMHC is expressed specifically in the heart and pulmonary myocardium [49]. Consistently, miR-208 is specifically and abundantly expressed in the heart, but we found a very weak expression (trace levels) in lungs (Figure 2A). These results are in agreement with the expression analysis of miR-208 in rats and humans [48]. Similarly, miR-499 is another intronic-derived miRNA located in the Myh7b gene (MHC 7b). miR-499 is abundantly and specifically expressed only in the heart and could not be detected in other tissues (Figure 2A). Similar observations have been reported for miR-499 in zebra fish [50].

#### miRNAs specifically or abundantly expressed in the liver

We observed specific and abundant expression of miR-122 in the liver (Figure 3B). Previous reports have characterized miR-122 as a liver-specific miRNA in diverse animals [51], and it has been linked to lipid metabolism and liver homeostasis [52]. We also found miR-194 abundantly expressed in the liver, and its level of expression was comparable with that of miR-122 (Figure 2B). The liver-associated expression of miR-194 in the mouse has been reported recently [53]. We also detected a trace amount of miR-194 in pig stomach (Figure 2B). miR-194 has been implicated in intestinal epithelial cell differentiation and maturation [54], and our finding of miR-194 expression in stomach of the pig is consistent with this previous report [54].

#### miRNAs abundantly expressed in the thymus

One of the key features of a functional immune system is its ability to distinguish antigens of foreign origin from those derived endogenously and subsequently to mount an immune response against such antigens. With respect to T cells, this goal is achieved through antigen recognition by T-cell receptors and a highly ordered developmental process in the thymus [55]. Many miRNAs are differentially regulated in hematopoietic lineages, and some have been shown to play roles in controlling the development of immune cells [55-57]. miR-142-3p, a hematopoietic-specific miRNA [56], exhibits distinct expression patterns during T-cell development and maturation [55]. Our study revealed miR-181 and miR-142-3p with relatively high expression in thymus (Figure 2C), and miR18a and miR-20a appeared to be weakly expressed in thymus (Figure 2D). miR-18a and miR-20a are located within the miR-17-92 cluster, which contains miRNAs known as "oncomiRs" because of their overexpression in many types of cancer cells [58,59]. Because these miRNAs are polycistronic, their similar expression patterns were expected. Surprisingly, the expression pattern of miR-20a's differed from that of miR18a in different tissues. These findings raise the possibility that the processing of mature miRNAs within a miRNA cluster may vary among tissues. Additionally, many other miRNAs, such as let-7, miR-98, miR-16, miR22, miR-26b, miR-29c, miR-30c and miR126, were also expressed abundantly in thymus (Figure 3).

#### miRNAs with highly varied expression patterns in different tissues

miR-143 expression varied substantially among the 14 tissues examined (Figure 3). It was highly expressed in stomach, testes, bladder and spleen and moderately in the other tissues but undetected in the thymus (Figure 2E). The expression of miR-378 was highly variable among the tissues tested (Figure 2E). The expression of miR-101 also varied among the tissues; it could be detected in liver, stomach, salivary glands, pancreas, spleen, lymph node and testes but not in thymus and bladder tissues. Some miRNAs, including miR-208, miR-101, miR-18a, miR-20 and miR-142-3p, showed a weaker expression than other miRNAs tested by small RNA blot analyses (Figures 2 and 3).

#### Ubiquitously expressed miRNAs

Some other miRNAs were detected in almost all tissues that we analyzed (Figure 3). let-7, miR-98, miR-130a and miR-16 showed uniform levels of expression in 13 different tissues but were hardly detected in pancreas (Figure 3A). miR-22, miR-26b, miR-29c, miR-30c and miR-126 exhibited almost similar expression patterns in all tissues examined (Figure 3B). However, a few minor differences were noticed in the expression of some miRNAs. For instance, miR-29c was expressed abundantly in stomach but only in trace amounts in thymus and ovary and miR-30c was relatively strongly expressed in heart, lungs, stomach and endometrium (Figure 3B).

## Discussion

Post-transcriptional gene regulation guided by miRNAs has emerged as one of the major gene regulatory mechanisms in higher eukaryotes. In the present study, we have identified a total of 120 conserved miRNA homologs in pig. Conserved miRNAs are likely play an important role in regulating basic cellular and developmental pathways from lower to higher organisms, whereas the non-conserved miRNAs are thought to be important in lineage-specific or species-specific pathways and functions [18].

### Small RNA blot analysis does not always reflect cloning frequency

Often, sequencing-based miRNA expression profiling has been used as a tool to measure the relative abundance of miRNAs [35,60]. Our sequence analysis in this study indicated that miR-1 family (miR-1a, miR-1b and miR-1c) has the highest abundance (411 sequence reads). In agreement with this observation, miR-1 is the most abundantly expressed miRNA in the heart but not in the liver or thymus (Figure 3), two other tissues used for miRNA library generation. A similar picture has emerged for miR-122, a liver-specific miRNA and one of the highly represented miRNAs in our sequences. However, this correlation may not always be the case. For instance, miR-133 is represented only by 4 clones (two reads each for 133a and 133b) in our sequences, which indicates a 100-fold lower expression level compared with that of miR-1 family, if cloning frequency taken as a measure of expression. However, our small RNA blot analysis indicated a different picture as miR-133 was detected as abundantly as miR-1 in the heart (Figure 2). These two miRNA genes – miR-1 and miR-133 – exist as a cluster and thus are always expressed together in mouse [42]. The discrepancies between the cloning frequency and small RNA blot results for miRNA-1 and miR-133 could not be attributed to the RNA source because the same RNA samples were used for both experiments (cloning and small RNA blot analysis). We also used approximately a similar amount (activity) of ^32^P-labelled probe for detection of miR-1 and miR-133. In our view, small RNA blot analysis provides a more reliable measure of abundance. Therefore, the above discrepancy could be largely due to biased cloning efficiencies of these two miRNAs. In Arabidopsis, a similar discrepancy between the cloning frequency and small RNA blot results was observed for miR398 previously [31]. What causes the biased cloning is not clear but could be attributed to the differences in the 5'- and 3'-end nucleotides, which are involved in ligating with the 5' and 3' adapters, respectively, or formation of secondary structures or adoption of a structure that prevents the exposure of 5' or 3' ends of the miRNA.

### Tissue-specific expression of miRNAs and their impact on gene expression

The expression of miRNAs is tightly regulated both in time and space. Tissue-specific miRNAs or a high level of expression of miRNAs only in certain tissues implies that these miRNAs play critical roles in the tissues where they are expressed. The observation that miR-22, miR-26b, miR-126, miR-29c and miR-30c are ubiquitously expressed in 14 different tissues of pig is interesting. Similarly, let-7, miR-98, miR-16 and miR-130a are abundantly expressed in 13 of the 14 tissues (except in pancreas) (Figure 3A). These results suggest a very important role for these miRNAs in the regulation of constitutive processes in diverse tissues.

The miR-17-92 cluster (polycistronic miRNA gene) encodes six miRNAs (miR-17, miR-18a, miR-19a, miR-20a, miR-19b-1, and miR-92-1) located in the third intron of a ~7-kb primary transcript known as C13orf25 [61]. Although miR-18a and miR-20a are likely derived from the same primary-transcript, the expression levels of these mature miRNAs are not similar (Figure 2D). This finding suggests that their processing varies in different tissues. Expression profiling studies have also revealed widespread overexpression of the miR-17-92 cluster in diverse tumor subtypes, including both hematopoietic malignancies and solid tumors such as those derived from breast, colon, lung, pancreas, prostate, and stomach [58,59].

Several miRNAs (miR-1, miR-133, miR-499, miR-208, miR-122, miR-194, miR-18, miR-142-3p, miR-101 and miR-143) have distinct tissue-specific expression patterns. Most animal miRNAs were thought to regulate several dozens of genes involved in diverse pathways and processes [62]. Additionally, many targets of these miRNAs are regulatory molecules such as transcription factors, which in turn may affect the expression of a large number of genes. Therefore, the impact of a miRNA expressed or not expressed in a specific tissue has a profound effect on the overall gene expression profiles in a particular tissue.

The use of three specialized tissues for library generation is a major limitation for the discovery of miRNAs in pig. Generating more small RNA libraries from diverse tissues should identify additional conserved and porcine-specific miRNAs. While our manuscript was in preparation, Kim et al., [29] reported 19 new pig miRNAs; 17 of these (let-7a, let-7b, miR-15a, miR-15b, miR-16, miR-17, miR-21, miR-23a, miR-23b, miR-24, miR-29a, miR-30b, miR-34a, miR-106b, miR-107, miR-130a, miR-140, miR-145, miR-152, miR-185, miR-199a, miR-210 and miR-221) are conserved miRNA homologs, and the remaining 2 are miRNA* sequences (miR-140* and miR-199b*). We have identified 11 of these miRNAs but did not find the remaining six miRNAs, possibly because these were not expressed or were expressed only at very low levels in the heart, liver and thymus tissues we used for the library.

## Conclusion

In summary, the present study has identified a total of 120 miRNAs in the pig. The expression for 22 conserved and three candidate miRNAs was determined in diverse tissues of the pig. The findings from this study will be a highly valuable resource for future transcriptomic and proteomic studies, as well as for annotating the pig genome in the near future.

## Methods

### Construction of miRNA libraries and sequencing

Tissues from 10 month-old crossbreed were collected and stored at -80°C by the DeSilva laboratory as part of the ongoing porcine gene expression project. Three tissues (heart, liver and thymus) used for the generation of small RNA library were obtained from one animal. The experiments were approved by the Institutional Animal Care and Use committees. Total RNA from different tissues was isolated with use of Trizol reagent (Invitrogen, Carlsbad, CA) following the manufacturer's instructions. The RNA pellet was dissolved in de-ionized formamide for effective denaturation of the RNA. Small RNAs of the desired size range (15–30 nt) were gel-purified by resolving in denaturing 15% polyacrylamide gel. These small RNAs were sequentially ligated with the 3' and 5' adapters as described in our previous reports [31-33]. Reverse transcription reaction was performed using the RT primer (AAGGATGCGGTTAAA), subsequently performing PCR using the reverse and forward (TACTAATACGACTCACTAA) primers. The purified PCR product using phenol/chloroform extraction and ethanol precipitation were shipped to OU's genomics facility for sequencing. Manufacturer instructions were followed for preparation of DNA samples for sequencing on the 454/Roche GS-FLX [63]. PCR products containing the 454 adaptors were quantified and diluted prior to amplification by emPCR [63]. After emPCR enrichment, the DNA was loaded onto a 454/Roche GE-FLX for massively parallel pyrosequencing.

### Sequence analysis and identification of new miRNAs

Our computational methods for analyzing 454 small RNA library was reported previously [64]. In brief, all small RNA reads without perfect matches to the most proximal 11 nt of both adaptor sequences were first removed. Reads corresponding to repeats were removed using the Einverted and Etandem programs in the EMBOSS package, respectively. The unique small RNAs were aligned to RepBase (version 13.04, obtained from ) and known non-coding RNAs (rRNAs, tRNAs, snRNAs, snoRNAs, etc., obtained from ) with NCBI BLASTN. In total, 304 small RNAs mapped to ncRNAs and 83 small RNAs mapped to RepBase were removed from further analysis. Then the small RNAs were mapped to the reported miRNAs in the miRBase (version 11), obtained from miRBase [65]. Small RNAs that matched known miRNAs of pig or other animal species resulted in identification of conserved miRNA homologs in pig. This analysis indicated that 120 unique small RNAs represented by 1562 reads matched with the conserved miRNAs listed in the miRBase.

### Small RNA blot analysis

Small RNA blot analysis was performed to determine the expression patterns of 22 conserved miRNAs. Total RNA (15–30 μg) was resolved on denaturing 15% polyacrylamide gel, along with labeled RNA markers. RNA was electrophoretically transferred to Hybond-N+ (Amersham) membranes, and the membranes were UV cross-linked and baked for 1 h at 80°C. DNA oligonucleotides complementary to small RNA sequences were end-labeled with γ-32P-ATP with T4 polynucleotide kinase (Invitrogen) used as a probe. Blots were pre-hybridized for at least 1 h and hybridized overnight with PerfectHYB Plus buffer (Sigma) at 38°C. Blots were then washed three times at 50°C and autoradiographed.

Note: While our manuscript is under review, two other papers have reported the identification of new miRNAs in pig using bioinformatics approach [66] and experimental approach [67]. Huang et al., [66] have bioinformatically predicted pig miRNAs and some of them were validated using micro array [66]. In another study, Sharbati-Tehrani et al., [67] cloned the small RNAs from pig but sequenced only small number of concatenated clones [30]. Sequence analysis has identified 11 miRNAs despite the fact that the sequencing depth is too low. Sharbati-Tehrani et al., [67] have reported 4 new miRNAs (miR-326, miR-423-3p, miR-484 and miR-451,) that could not be identified in our study, which could be attributed to the use of very specialized tissues (Jejunium, spleen, ileum and kidney) for their study.

## Authors' contributions

RS designed the research, coordinated the project and wrote the paper. AMR performed the expression analysis and GJ constructed the small RNA library. YZ and WZ performed the computational analysis and wrote the corresponding part. SM, GW and BR sequenced the small RNA library. UD provided the pig tissues used in this study. All authors read and approved the final manuscript.

## Supplementary Material

Additional file 1 **Processed procine small RNA sequences obtained using the 454 pyrosequencing.** Excel spreadsheet displaying Processed procine small RNA sequences obtained using the 454 pyrosequencing. Click here for file
